# Environmental Enrichment in Rabbit Husbandry: Comparative Impacts on Performance and Welfare

**DOI:** 10.3390/ani14162367

**Published:** 2024-08-15

**Authors:** Karim El-Sabrout, Anjum Sherasiya, Sohail Ahmad, Sarah Aggag, Eleonora Nannoni, Damiano Cavallini, Giovanni Buonaiuto

**Affiliations:** 1 Poultry Production Department, Faculty of Agriculture, Alexandria University, Alexandria 21545, Egypt; 2 Veterinary World, Wankaner, Star, Gulshan Park, Wankaner 363621, India; 3 Poultry Production Department, Faculty of Animal Production and Technology, University of Veterinary and Animal Sciences, Lahore 54000, Pakistan; 4 Genetics Department, Faculty of Agriculture, Alexandria University, Alexandria 21545, Egypt; 5 Department of Veterinary Medical Sciences, University of Bologna, 40064 Bologna, Italydamiano.cavallini@unibo.it (D.C.)

**Keywords:** behavior, environmental stress, housing enrichment, productive performance, rabbit, sensors, welfare

## Abstract

**Simple Summary:**

Rabbit meat is consumed in both developing and developed countries. Intensive animal housing systems often subject animals to challenging environmental conditions, resulting in behavioral changes and the activation of stress responses at the neurological and hormonal levels, adversely affecting their health and productivity. Environmental enrichment strategies aim to mitigate these impacts by facilitating animals’ adaptation to their farming environment, thereby reducing stress. This review comprehensively examines the efficacy of various environmental enrichments, whether implemented individually or in combination, in enhancing the welfare and performance of rabbits within intensive farming settings. Furthermore, this review discusses future developments and the integration of precision rabbit farming approaches aimed at further improving animal welfare.

**Abstract:**

Rabbits are highly susceptible to environmental stress. Such stress, stemming from conventional housing conditions, can negatively impact well-being and productivity. Some of these negative consequences are increased susceptibility to diseases and infections and reduced growth rates and reproductive performance, as well as increased behavioral issues such as aggression. Environmental enrichment is the modification of the environment in which a domesticated animal lives in order to meet the species’ behavioral needs. The objective of providing enrichment is to facilitate animals in expressing natural behaviors, thereby preventing behavioral frustration and negative affective states. Several inexpensive and safe materials can be used to enrich rabbit enclosures. This review emphasizes the significance of implementing diverse environmental enrichments to alleviate stress in rabbit farming. It summarizes their effects on animal performance and welfare while exploring potential future perspectives in this field.

## 1. Introduction

The global consumption of rabbit products is increasing due to their high nutritional value and affordable prices [1]. These meats offer essential proteins, vitamins, and minerals, and are generally lower in fat compared to red meats. As demand increases, the sector faces challenges in maintaining sustainable and ethical production practices. Over the past few years, consumers have expressed great interest in improving animal welfare and product quality [2]. Prioritizing animal welfare aligns with ethical principles, leading to increased consumer satisfaction and long-term benefits for both animals and consumers.

In modern intensive husbandry systems, animals can be exposed to challenging farming conditions resulting in stress responses, abnormal behavior, body lesions, or more severe consequences. The rabbit industry is not immune to this issue. Decades of genetic selection aimed at increasing productivity have resulted in rabbits with poor resilience to stress and a lower capability to cope with suboptimal farming conditions, negatively impacting their health, welfare, and productivity [3,4]. The negative consequences include an increased susceptibility to diseases and infections and reduced growth rates and reproductive performance, as well as increased behavioral issues such as aggression and stereotypies. Housing conditions are the most important factor influencing the welfare of raised rabbits. Thus, establishing better living conditions that align with animal welfare concerns is important [5]. Wire cages, overcrowding, a lack of space availability, poor ventilation, inadequate lighting, and limited opportunities to carry out natural behaviors are some of the most prominent stressors in conventional housing systems [5,6]. There are some strategies and tools that can help reduce environmental stress, creating a more enriched and comfortable environment for rabbits, and promoting better animal welfare and performance.

**Figure 1:**
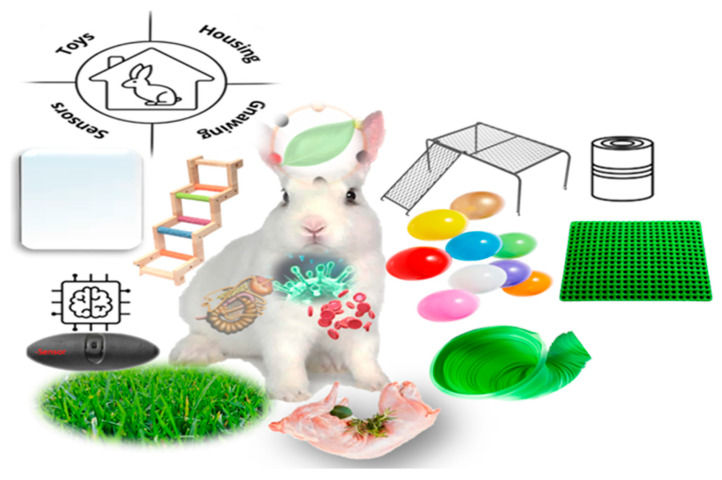
Potential enrichment materials to improve rabbit health and productivity.

Environmental enrichment is a key aspect of animal welfare, particularly for small livestock like rabbits. It could be defined as the modification of the environment in which a domesticated animal lives in order to meet the species’ behavioral needs [7]. It involves adding physical and social stimuli to the living environment to enhance the animals’ mental and physical health [7,8]. Several inexpensive and safe objects and tools can be used to enrich rabbit environments, such as toys, mirrors, hiding places, and tunnels [7,9] (Figure 1). In some regions, such as Europe, the use of environmental enrichments is regulated by law for certain categories of livestock, like pigs [10] and rabbits [11]. On the other hand, some other countries have no regulations specifically addressing the protection and welfare of rabbits in farming according to the European Food Safety Authority (EFSA) [5].

Increasing animals’ behavioral choices to promote abilities has a crucial effect on animal welfare as well as reducing stress and boredom [7,12]. Environmental enrichment can improve animal welfare, prevent abnormal behavior, and generate feelings of comfort, pleasure, interest, and a sense of control by allowing animals opportunities to engage in rewarding behaviors, which can include feeding, exploration, standing, foraging, resting, and social interactions [8,12]. Additionally, environmental enrichment has the potential to promote animals’ cognitive development, resource utilization, and adaptability [13,14]. These potential benefits are particularly relevant considerations in modern commercial rabbit production systems. Therefore, this review delineates the importance of using different environmental enrichment materials, either individually or in combination, to improve the performance and welfare of rabbits.

Additionally, this integrated review provides educational and practical synergy; farmers and researchers benefit from a broader understanding of enrichment techniques that can enhance the productivity and welfare of rabbits. This holistic perspective can lead to more informed decision-making and policy development. Finally, implementing enrichment strategies that are effective for multiple species can contribute to the economic and environmental sustainability of farming practices. By reducing costs and minimizing environmental impact, these practices support more sustainable agricultural systems.

## 2. Environmental Stress on Rabbits

Stress is an adaptive phenomenon in animals, occurring in response to environmental changes [15]. This process involves the organism’s reaction to a stimulus that activates the hypothalamic–pituitary–adrenocortical (HPA) axis and the sympathetic–adrenomedullary system [16]. Rabbits are sensitive social animals that are always raised in cages. Does (females), for example, are generally kept in individual cages [17]. It is important to note that this situation varies widely depending on farm management practices or specific legislation. Although rabbits are typically raised in conventional and/or enriched cages, they can also be raised in indoor parks and outdoor systems [5]. 

Environmental stressors pose serious threats to animal welfare and production [14,18]. The adverse effects of environmental stress on small livestock animals, such as rabbits, can manifest in poor overall performance, including a reduced feed intake, growth, and reproduction, as well as increased mortality rates. Additionally, stress profoundly impacts the genetic structure of animals, like rabbits, leading to detrimental effects on their DNA integrity [18,19,20,21,22]. Although the immediate consequences of stress on behavior and physiology are well documented, the long-term effects of stress on the genetic composition of animals, particularly rabbits, are often overlooked. This essay delves into the intricate relationship between stress and genetic structure, shedding light on how chronic stress can inflict damage at the molecular level. When animals encounter stressors, whether from environmental factors or social interactions, their bodies initiate a cascade of physiological responses to cope with the perceived threat [23,24,25,26]. The release of stress hormones, such as cortisol and adrenaline, triggers a series of adaptive changes aimed at enhancing survival [16]. However, prolonged exposure to stress disrupts this delicate balance, leading to dysregulation in various biological systems [27].

The profound impact of chronic stress on the genetic structure of animals has been well documented [20,26]. Previous studies have shown that stress can induce alterations in gene expression, affecting crucial biological processes, such as immune function, metabolism, and reproduction. Additionally, stress-induced changes in DNA methylation patterns have been linked to the increased susceptibility to diseases and reduced overall fitness of these animals [20,21]. One of the most alarming consequences of chronic stress is the DNA damage of animals [19,21]. Oxidative stress, a consequence of prolonged exposure to stress hormones, can cause DNA strand breaks and mutations, compromising the stability of the genome [22]. DNA damage not only accelerates aging but also increases the risk of developing genetic disorders and hereditary diseases. In conclusion, the complex interplay between stress and genetic structure highlights the need for a holistic approach to animal well-being. By understanding the genetic consequences of chronic stress on animals like rabbits, we can pave the way for innovative research and targeted interventions aimed at preserving genetic integrity and promoting resilience in the face of environmental challenges.

## 3. Environmental Enrichment for Rabbits

Given the prevalence of welfare issues in some rabbit farming systems, it is evident that enrichment plays a crucial role and should be implemented on a large commercial scale to help rabbits better adapt to their farming environment and offer them additional space to express their natural behaviors. 

Table 1 depicts some of the most recent studies on the effects of environmental enrichment on rabbits.

Hansen and Berthelsen [28] studied the impact of keeping rabbits in enriched cages, with access to shelter and increased cage height, on their behavior and welfare. Rabbits raised in conventional cage systems exhibited excessive self-grooming, restlessness, bar-gnawing, and stress than rabbits raised in an enriched cage system. These results indicate that rabbits kept in an enriched cage system, particularly females, had better welfare than those kept in a conventional cage system due to access to shelter and improved opportunities to interact with their surroundings. Hansen and Berthelsen [28] reported that access to shelter and the presence of elevated platforms (or high shelves) should be considered environmental enrichment materials in rabbit cages. The results of this study support the concept that environmental enrichment can reduce abnormal activity and stress, particularly in female rabbits.

Trocino et al. [29] evaluated the use of two types of enrichment (an elevated plastic slatted platform and/or a plastic hiding tube) on the behavior and productive performance of growing rabbits housed in collective pens in large groups. According to their findings, using the platform allowed rabbits to move up and down, rest in a more comfortable position, and increase their exploratory behavior, while maintaining production performance. However, more injured rabbits were found at the end of the study in the pens with platforms. The inclusion of the tube did not change the behavior but reduced the growth performance. Hence, the elevated platforms acted as a useful structural enrichment for animal behavior, but negatively impacted injuries; therefore, the effectiveness of the tube was not confirmed [29].

Adding bedding material on the wire net of the cage floor for environmental enrichment may improve animal husbandry and welfare [30,31]. On the other hand, Feng et al. [32] examined the effects of using a willow stick, a rubber duck, and a can of beans on rabbit behavior and serum hormonal levels. They found that enrichment with a can of beans was the most suitable for rabbits’ behavior and health, among the three tested. Working with cardboard and rubber objects as potential environmental enrichment materials for rabbits, Da Silva et al. [45] reported no significant differences in behavior among the treatment groups. One possible reason for the lack of changes in rabbit behavior after receiving gnawing objects is that all the animals were provided with hay during the experiment. According to Berthelsen and Hansen [33], hay can be regarded as a source of enrichment for rabbits. Huang et al. [34] evaluated the effects of a scratching card, gnawing material, and a platform on female rabbit behavior. They reported that the does preferred plastic mesh flooring over wire mesh flooring. Furthermore, providing these enrichments stimulates specific behaviors, such as gnawing, scratching, and rearing up, which may enhance their welfare. Princz et al. [35] reported that growing rabbits prefer plastic net floors and cages provided with gnawing sticks. Additionally, the presence of gnawing sticks decreased the frequency of physical injuries. Rashed and El-Edel [36] assessed the effect of different floor types (plastic, wire, or combination) on the behavior, welfare, and productivity of growing rabbits. They found that a plastic floor, either alone or combined with wire, significantly increased exploration, walking, and body care behaviors compared to wire flooring. Feeding behavior was significantly higher in rabbits reared on a combination floor than in other groups, whereas drinking behavior was significantly higher in rabbits reared on a plastic floor than in other groups. Conversely, resting behavior was significantly higher in rabbits reared on wire flooring. The authors also observed that the rabbits showed a higher preference for plastic flooring over wire or combination flooring. Furthermore, the body weight and daily weight gain for the group reared on plastic floors were significantly higher than those on wire or combination floors. Rashed and El-Edel [36] concluded that the presence of a plastic net floor increased the productivity and welfare of rabbits. Similarly, Gharib et al. [46] noticed that placing plastic or rubber mats on the floor of a wire cage reduced aggressive and abnormal behavior, lowered cortisol levels, and improved the productivity and welfare of growing rabbits.

Enriching rabbit cages with toys or gnawing materials could provide a better farming condition for rabbits’ teeth health since these materials are more abrasive, thus preventing medical concerns that could jeopardize their well-being [7]. The presence of toys in cages has a substantial impact on rabbit behavior, as rabbits with toys spend significantly more time chewing than those without toys. Thus, objects, particularly rubber ones, can enrich rabbit cages and improve animals’ welfare. Furthermore, using different cage enrichments, such as rubber floors, plastic colored balls, and mirrors, has no adverse impact on the cecal bacteria counts of growing rabbits [9].

Mirrors have recently become a popular enrichment material for rabbit cages. These appear to mimic a situation where rabbits are permitted to make eye contact with other conspecifics, which increases natural activities, including olfactory investigation [30]. Mastellone et al. [37] found that the use of mirrors may represent a low-cost, efficient means to stimulate the expression of natural behaviors in rabbits bred in small groups in a free-range system. Rabbits raised in mirrored cages with visual and olfactory contact expressed much higher levels of natural activities, such as olfactory investigation and allo-grooming activity, than those in the other groups. Musco et al. [38] reported that mirrors can have positive effects on growth performance and carcass quality traits. They found that rabbits raised in cages enriched with mirrors showed the best feed conversion rate and dressing percentage. The use of mirrors can improve the rabbit’s growth performance and carcass traits by lowering the rabbit’s locomotion activity in comparison to the other tested systems. Additionally, Dalle Zotte et al. [39] showed that rabbits preferred the cage half-enriched with mirrors, which reduced marginally with age. However, the presence of conspecifics did not diminish this interest in mirrors at varying stocking densities. The findings of Dalle Zotte et al. [39] imply that the presence of mirrors provides benefits, may be connected to comfort and welfare, and thus might be used as environmental enrichment for fattening rabbits and, in particular, for rabbits housed individually for prolonged periods. Likewise, Edgar and Seaman [40] reported that when single housing cannot be avoided, mirrors might be appropriate to partially compensate for social contact in female rabbits. However, adding multiple mirrors in rabbit cages, particularly for a long time, without monitoring rabbit behavior and reactions, especially at young ages (prior to weaning), may result in abnormal behavioral effects, such as anxiety and fear [47].

According to the literature, sensory stimulation in the form of olfactory, auditory, and visual cues can be used as an environmental enrichment strategy for caged animals [48]. Environmental enrichment appears to be associated with an increased frequency of several natural behaviors, including allo-grooming, a social behavior involving mutual recognition and pheromonal olfactory stimulation. According to Luzi et al. [41], a lack of stimulation during rabbit fattening may result in welfare issues. They also reported that providing fattening rabbits raised in colony cages with environmental enrichments such as a wood stick hanging down from the cage ceiling could enhance their biological functioning, increase their growth rate without worsening their health status, allowing the animals to perform a broader range of specific behaviors and reducing the occurrence of stereotypic behaviors. Similarly, Zucca et al. [42] stated that engagement with an object, such as a wooden stick, allows the rabbit to carry out exploratory behavior, especially in small-sized groups. According to Buijs et al. [43], the addition of a wooden structure enhances welfare, reducing lateral lying and interactions with conspecifics. Despite social contact often being regarded as beneficial, rabbits could have used the structure as a visual or physical barrier to avoid undesired contact with conspecifics. Bozicovich et al. [44] investigated whether two eucalyptus sticks suspended from the cage ceiling affected the behavior and relative brain weight of growing rabbits. They found that whereas enrichment had no effect on growth performance, the males in enriched cages had heavier brains than those in non-enriched cages. Furthermore, the rabbits kept in enriched cages had fewer wounds on their skin, indicating lower aggression levels.

Finally, it is important to remember that any application of environmental enrichment undoubtedly impacts the economic sustainability of the farm and, consequently, the entire production sector, even though this consideration is not addressed in the previously cited studies. The primary limitation in adopting environmental enrichments and better technologies generally depends on the individual choices and preferences of rabbit farmers, primarily driven by economic benefits. As reported by Mondin et al. [49], rabbit farmers choose to adopt higher animal welfare standards if they gain advantages such as a higher market price often due to better meat quality, as noted by de Greef et al. [50], or reduced costs [51]. Like other sectors, rabbit farming is influenced by market dynamics, which generate high price volatility, followed by a decline in consumption in certain areas. These factors affect all farmers uniformly [52,53,54]. Therefore, the ability of farmers to adopt any innovation in animal welfare largely depends on the impact of such innovation on farm profitability.

However, it is important to note, as observed by Dockès and Kling-Eveillard [55], that sometimes farmers are also influenced by non-economic motivations, such as societal incentives, the social image of the profession, and belonging to a group of producers [56,57]. Health-related reasons can also influence farmers’ decisions, encouraging them to invest in infrastructure and adopt measures to improve the welfare of their animals, as reported by Le Bouquin et al. [58]. Nevertheless, regardless of the state of the economy or the societal structures in place, the well-being of animals must always be a priority and the welfare of captive animals, in particular, must be guaranteed.

## 4. Future Perspectives

In addition to environmental enrichment materials, enhancing the welfare of rabbits also involves optimizing the use of existing facilities. Although the application of precision livestock farming technologies has not yet achieved widespread success among rabbit breeders, the deployment of these technologies in animal husbandry—such as digital imaging systems, vocalization analysis, thermal/infrared analysis, and Raman spectroscopy—could play a crucial role [59]. These technologies enable efficient and continuous data collection from multiple sources to monitor animal behavior, health, and production. They can be instrumental in detecting behavioral changes, physiological issues, and illnesses in rabbit farms [60]. By analyzing environmental and animal-based data, these tools help farmers make informed management decisions and implement targeted interventions to address the identified issues. Sensors, for example, play a crucial role in collecting data on daily animal behavior and physiological parameters (e.g., feeding behavior, body weight, or activity) [61]. By tracking these metrics, farmers can gain valuable insights into the welfare of their animals and choose the best environmental enrichment approach. On the other hand, artificial intelligence (AI) technology can analyze vast amounts of data collected by monitoring systems to predict trends, identify patterns, and optimize farm operations [61]. AI algorithms can help breeders and producers make informed decisions regarding feed management, ventilation/lighting control, and disease prevention. By using AI, breeders can also enhance efficiency, minimize resource wastage, and improve overall farm performance. Moreover, data collected using these tools can also be utilized for the genetic improvement of livestock species, including rabbits. This contributes to the selection of better-performing animals that are better adapted to their farming environment, as reported by Negretti et al. [62].

To date, the most widespread technology used in small livestock farms are systems for monitoring air temperature, velocity, humidity, and levels of harmful gases, such as ammonia [59,63]. This prevalence is due to the significant impact of environmental conditions, particularly temperature and relative humidity, on both the physiological state and productivity of animals [64]. Rabbits, in particular, are sensitive to heat, having few sweat glands and difficulty dissipating body heat [65,66]. For this reason, several researchers have studied systems to monitor thermal stress conditions and general changes in body temperature. Changes in the body surface temperature, as a consequence of both acute and chronic stress, can be detected using infrared thermography (IRT) cameras for temperature measurement [67,68]. An infrared camera measures electromagnetic energy and detects different wave frequencies emitted by each temperature value (Stewart et al., 2005) [67]. The main strength of IRT is that it is a non-invasive and non-contact method for measuring the surface temperature of animals [69]. In rabbits, infrared thermography has been utilized in various studies (e.g., [68,70]). Ludwig et al. [71] and Luzi et al. [72] were the first to use this technology to monitor the temperature of specific body areas (eye bulb, periocular area, and ear skin) in rabbits subjected to thermal stress. Their work yielded promising results and paved the way for subsequent studies. Another example of this technology’s application is provided by researchers who utilized the same principles, reported by the authors of [71,72], to assess heat stress in does and bucks when temperatures exceeded the thermoneutral zone of rabbits reared under typical commercial conditions. These authors compared results obtained from different body areas of the rabbit (eyes, ears—both externally and internally—and nose), reporting that the ocular temperature exhibited a narrower range of variation compared to other body areas, and highlighting challenges in capturing clear images of the internal ear. They suggested that the minimum eye temperatures and maximum or minimum nose temperatures could be used to assess heat stress in rabbits. De Lima et al. [68] employed infrared thermography (IRT) to detect differences in the surface body temperature of breeding rabbits exposed to two distinct temperature conditions above their comfort zone. One condition involved temperatures below the combination of temperature and humidity considered severely stressful for rabbits, while the other condition involved several hours per day of exposure to temperatures ranging from severe to very severe stress. This study is particularly interesting as it investigates whether IRT can highlight if animals are subjected to more severe stress in one environment by detecting higher heat losses. A particularly interesting application of infrared thermography (IRT) is demonstrated by Vadlejch et al. [73], who used this technology to monitor changes in skin temperature in rabbits during coccidiosis infection. Their study illustrates how IRT can be employed not only to monitor stress but also to predict and detect diseases. 

The continuous monitoring of these factors enables farmers to detect potential issues early and implement proactive measures to maintain optimal conditions for the animals. However, the potential of sensor technology in the sector is much broader, encompassing technologies such as ultra-wideband (UWB) tracking, computer vision (CV), accelerometers, radio frequency identification (RFID), and the use of artificial intelligence for data analysis [74,75].

As previously mentioned, precision livestock farming technologies are not widely used in rabbit farming. This is likely due to the fact that, unlike other sectors (such as dairy cattle or laying hens), rabbit farming is a “niche” sector with a lower turnover. Table 2 presents several studies on the application of various technologies in rabbit farming [4,68,70,71,72,73,76,77,78,79,80,81,82,83,84,85,86,87,88]. In general, the most widespread technology in rabbit farms today concerns environmental monitoring. The characteristics of rabbit farms and the species’ sensitivity to respiratory problems make the monitoring of farming conditions—such as temperature, humidity, and air quality—particularly important. This monitoring is crucial for preventing health issues that can impact both production and animal welfare. An example of such an application is reported by Zhang and Qian [78], who designed a technology-based intelligent farm environment monitoring system. Their goal was to address the limitations of existing wired monitoring systems. Using rabbits as a case study, they investigated the main environmental factors affecting rabbit growth. The system monitors key environmental parameters, including temperature, humidity, light, and ammonia levels. An additional contribution is provided by Yingdong and Wenshen [80], who proposed a real-time environmental monitoring system for rabbit houses based on Narrowband Internet of Things (NB–IoT). Their system aimed to overcome the limitations of traditional wired networks, reducing network costs and circuit component expenses. An Arduino development board and the Quectel BC260Y–NB–IoT network module were used, along with the message queuing telemetry transport (MQTT) protocol for remote telemetry transmission, enabling network connectivity and communication with an IoT cloud platform. Multiple sensors, including SGP30, MQ137, and 5516 photoresistors, were integrated into the system to achieve the real-time monitoring of various environmental parameters within the rabbit house, such as sound decibels, light intensity, humidity, temperature, and gas concentrations [80]. The collected data were stored for further analysis and could be used to inform environmental regulation and monitoring in rabbit houses, both locally and in the cloud. Signal alerts based on circuit principles were triggered when thresholds were exceeded, creating an optimal living environment for the rabbits. 

One particularly interesting technology that is relatively easy to deploy in small livestock farming is image processing technology [80]. This technology involves capturing the movement patterns in animals, allowing for the measurement of their activity and behavior, which can be used as an indicator of their welfare. Currently, there are no studies reporting the application of this technology to rabbit farming, likely due to the specific nature of this type of livestock. However, some studies have evaluated the use of infrared video technology to assess doe–kit contact [83]. This parameter is crucial as it directly influences both the kits’ quality of life and the breeder’s profitability [89]. Monitoring systems for the litter can provide precise reports to the breeder on the number of times the doe enters the nest to nurse the kits, allowing for timely intervention in case of issues. An additional development of this technology could involve directly monitoring the litter. In the first days of life, kits are particularly sensitive to temperature fluctuations [90]. Therefore, this technology could be used to monitor the nest temperature, helping to reduce kit mortality. In short, this technology offers advantages over conventional farming issue detection methods like visual inspection, because it allows for earlier treatment or corrective actions to prevent further spread of infection among the animals and reduce mortality.

Another promising precision livestock farming (PLF) technology that could be easily applied in the commercial rabbit sector is sound analysis. This method can recognize specific vocalizations and use them as indicators of both positive (e.g., social calls) or negative (e.g., coughing) conditions. However, no studies are currently available in the literature on this topic. Nevertheless, Ilès [91] observed changes in vocalizations in rabbit does exposed to bucks, which were related to their reproductive state (pregnant or not). The conclusions of this study suggest that analyzing vocalizations could be used as an indicator of pregnancy in domestic rabbits. Further research is desirable, as the current method for diagnosing pregnancy in does involves manual palpation by an operator. This practice can be very stressful for rabbits, as they may associate handling with a predation attempt.

Currently, the market offers various robotic systems, typically autonomous vehicles or devices that travel on tracks, such as those applied in some advanced poultry farms [92]. These robots contribute to improving the quality of bedding and air, and their movement encourages physical activity among the animals, positively impacting their health [61,93]. According to Jiang et al. [88], robotics can also be used in the distribution of feed, helping to reduce stress, particularly in species such as rabbits that are highly susceptible to loud noises. Additionally, these technologies can be employed in implementing precision feeding protocols [84,88,94]. Thus, the integration of monitoring systems, artificial intelligence, sensors, and environmental enrichment approaches is crucial for enhancing farming conditions in rabbit farms. By leveraging these technologies and practices, farmers can improve animal welfare, optimize farm productivity, and mitigate environmental impact. Furthermore, it is imperative for the rabbit industry to embrace further research and investigation, notably on diverse environmentally friendly enrichment tools, to ensure sustainability, animal well-being, and production efficiency.

## 5. Conclusions

The intensive nature of most rabbit farm practices can lead to environmental challenges and impact animal welfare. Enhancing rabbits’ quality of life is a crucial aspect, as it impacts welfare, health, and production. This review updates readers and farmers on different environmental enrichment strategies, such as using toys, mirrors, and tunnels for rabbits to improve their behavior and performance. Although some farmers remain hesitant to adopt these strategies on their farms, the well-being of animals must always be a priority, regardless of economic conditions or societal structures. These strategies can be applied separately or in combination and could be part of a farm management system provided with sensors for the early detection of potential problems and to take proactive measures to ensure that animals are kept in optimal conditions. 

## Figures and Tables

**Table 1:** Main recent studies on environmental enrichment in rabbit farms.

Type of Enrichment	Housing	Reference
Shelter and raised height	Group cage	Hansen and Berthelsen [28]
Platform and tube	Collective pen	Trocino et al. [29]
Wire net and straw litter	Collective pen	Jekkel and Milisits [30]
Straw litter	Group cage	Siloto et al. [31]
Willow-stick, rubber duck, and bean can	Group cage	Feng et al. [32]
Hay as gnawing material	Group cage	Berthelsen and Hansen [33]
Platforms and gnawing materials	Group cage	Huang et al. [34]
Gnawing sticks	Group cage	Princz et al. [35]
Gnawing sticks	Group cage	Rashed and El-Edel [36]
Mirrors	Group cage	Mastellone et al. [37]
Mirrors	Group cage	Musco et al. [38]
Mirrors	Group cage and pen	Dalle Zotte et al. [39]
Mirrors	Single cage	Edgar and Seaman [40]
Wooden stick	Colony cage	Luzi et al. [41]
Wooden stick	Group cage	Zucca et al. [42]
Shelter	Open-top wire cages	Buijs et al. [43]
Wooden stick	Group cage	Bozicovich et al. [44]
Plastic colored balls	Group cage	Elsayed et al. [9]

**Table 2:** Some studies on the application of precision farming technologies in rabbit farms.

Reference	Type of Technology	Measured Output
Ludwig et al. [71]	Infrared thermography	Thermal stress condition
Luzi et al. [72]	Infrared thermography	Skin temperature changes
Rafael et al. [70]	Infrared thermography	Skin temperature changes
De Lima et al. [68]	Infrared thermography	Body temperature and heat losses
Vadlejch et al. [73]	Infrared thermography	Parasite infection
Jaén-Téllez et al. [4]	Infrared thermography	Body temperature
Chen et al. [76]	Telemetry	Body temperature
Navarrete et al. [77]	Telemetry	Body temperature, activity
Zhang and Qian [78]	Wireless sensor networks	Environmental monitoring
Noor et al. [79]	PIC microcontroller	Automatic feeding system
Yingdong and Wenshen [80]	Internet of Things	Environmental monitoring
Mora et al. [81]	Accelerometer	Activity
Ma et al. [82]	Ultra-wideband radar	Respiratory frequency
Schud et al. [83]	Infrared video technology	Doe–kit contact
Sánchez et al. [84]	Electronic feeder	Feeding behavior
Psiroukis et al. [85]	Thermal imaging	Localization
Adedeji et al. [86]	Deep learning	Behavior
Ipek et al. [87]	Computer vision	Behavior
Jiang et al. [88]	Robotics	Automatic feeding

## Data Availability

The supplementary data can be made available from the corresponding author upon reasonable request.
